# Proximate Composition, Amino Acid Content and Antioxidant Activity of Different Potato Varieties from the Matese Plateau (Southern Italy)

**DOI:** 10.3390/foods15101634

**Published:** 2026-05-08

**Authors:** Nicola Landi, Sara Ragucci, Sofia Del Gaudio, Maria Giuseppina Campanile, Robina Khan, Maria D’Angelo, Stefania Papa, Enrica De Falco, Antimo Di Maro

**Affiliations:** 1 Institute of Crystallography, National Research Council, Via Vivaldi 43, 81100 Caserta, Italy; 2 Department of Environmental, Biological and Pharmaceutical Sciences and Technologies (DiSTABiF), University of Campania ‘Luigi Vanvitelli’, Via Vivaldi 43, 81100 Caserta, Italy; sara.ragucci@unicampania.it (S.R.); mariagiuseppina.campanile@unicampania.it (M.G.C.); robina.khan@unicampania.it (R.K.); maria.dangelo@unicampania.it (M.D.); stefania.papa@unicampania.it (S.P.); 3 Department of Pharmacy (DIFARMA), University of Salerno, Via Giovanni Paolo II 132, 84084 Fisciano, Italy; sodelgaudio@unisa.it (S.D.G.); edefalco@unisa.it (E.D.F.)

**Keywords:** antioxidant capacity, amino acids, food quality, Matese plateau, nutritional components, *Solanum tuberosum* L.

## Abstract

Here, the proximate composition, total amino acid content and antioxidant activity of Agria, Désirée and Kennebec potato varieties cultivated on the Matese plateau (Campania region, Southern Italy) were evaluated. Significant differences were observed among varieties in terms of proteins (1.98–3.07 g/100 g FW), carbohydrates (12.05–15.78 g/100 g FW) and moisture (78.42–84.68 g/100 g FW), while lipids were consistently low (~0.1 g/100 g FW), ~2.6-fold lower than ‘gold’ potatoes, used as a reference. Ashes were relatively high (1.10–1.39 g/100 g FW), ~1.4-fold higher than ‘gold’ potatoes. Total amino acid profiles were similar, although statistically significant differences were observed for Glx (glutamic acid + glutamine) and Asx (aspartic acid + asparagine), which are the most abundant amino acids, followed by valine, arginine and lysine. The chemical score of essential amino acids highlights that Matese potato varieties have a high nutritional content of phenylalanine + tyrosine and threonine, with average chemical scores of ~99.8% and 91.6%, respectively, while leucine is the limiting amino acid. The free amino acid profile does not show statistically significant differences. The total phenolic content (TPC) of analysed varieties (57.85–123.27 mg GAE/100 g of FW) was higher than those reported in the literature and directly correlated with the evaluated antioxidant activity (ABTS and DPPH). Finally, Matese potatoes are rich in potassium, phosphorus, calcium and magnesium, with minor minerals (~1.6%) and selenium traces (~0.53 µg/100 g FW). Overall, these findings highlight the potential of Matese potatoes to enhance local consumption, preserve culinary heritage and support gastronomic tourism growth.

## 1. Introduction

Potatoes (*Solanum tuberosum* L.) are the world’s fourth most important and widespread food crop after rice, wheat and maize [1,2]. This tuber originated in the Andes Mountains of South America, specifically in Peru and Bolivia, where indigenous people began domesticating wild potato plants around 8000–10,000 years ago, while archaeological evidence dates its cultivation back to 8000–5000 years BC near Lake Titicaca [3,4]. Spanish conquistadors discovered potatoes in Peru during the 1530s and introduced them to Europe around the late 16th century. They were initially popular in Spain and Ireland, before spreading more widely across the continent [5,6]. In the 18th century, potatoes became a staple in Europe, contributing to population growth due to their high yield and nutrition [4,7].

Potatoes are underground tubers that develop from the stems of *S. tuberosum* plants belonging to the Solanaceae family. The plant has green leaves and can produce small toxic fruits, like other nightshades [8], while the tuber is edible [9,10]. There are thousands of potato varieties, differing in size, colour (white, yellow, red and purple) and starch content: waxy for salads and starchy for mashing [11].

Potatoes are an excellent source of carbohydrates (~17 g per 100 g of fresh weight; FW), mostly starch (~15 g per 100 g FW), which provides readily available energy [12,13]. They also contain moderate amounts of high-quality proteins (~2.0 g per 100 g FW) with a favourable amino acid profile, particularly rich in lysine [14]. Potatoes are naturally low in fat (~0.1 g per 100 g FW) and cholesterol-free, making them suitable for healthy diets [15]. Indeed, minimally processed potatoes contribute substantially to daily micronutrient intake, being a significant source of essential vitamins and minerals, including vitamin C, vitamin B6, potassium, magnesium, phosphorus and iron [12]. Potato dietary fibre (~2.2 g per 100 g FW) is mostly present in the peel, promoting digestion and helping to regulate glucose blood levels [13,16]. In addition to their considerable nutritional composition, potatoes possess several bioactive compounds with nutraceutical benefits [14]; among them, phenolic acids (e.g., chlorogenic acid), flavonoids, carotenoids and anthocyanins are particularly present in coloured-flesh potato varieties [17]. These compounds exhibit antioxidant, anti-inflammatory and antimicrobial properties, helping to reduce the oxidative stress and the risk of chronic diseases, such as cardiovascular disorders, diabetes and cancer [14,18]. Resistant starch in potatoes also acts as a prebiotic, promoting gut health and improving insulin sensitivity [19]. The growing interest in potatoes as a functional food highlights their potential role in preventive nutrition and health promotion [20,21].

In Italy, considering the peculiar pedo-climatic condition and given the remarkable adaptability of the *S. tuberosum* plant, potato cultivation has spread throughout its territory over time [22]. On the other hand, the unique characteristics of some territories have allowed for the selection of local cultivars that are highly appreciated by consumers [23]. In addition, in several cases, Italian potato cultivation is closely associated with mountain farming [22]. Indeed, local populations have always cultivated potatoes in mountainous territories and neighbouring valleys, in order to provide food reserves during winter as a safeguard against famine [24,25]. Furthermore, the cultivation of potatoes in mountainous territories guarantees products of high quality and limits preventive or phytosanitary treatments [26,27]. Indeed, the altitude and light exposure of the fields, combined with large daily temperature swings (day/night) [28], can prevent or reduce pathogen diseases, while biennial crop rotation guarantees the availability of minerals in the soil and reduces reliance on organic or chemical fertilisers [29,30,31]. Environmental conditions such as altitude, temperature, amplitude and soil composition are known to influence the biochemical and nutritional composition of potato tubers [26]. Temperature regimes during tuber development influence enzyme activities involved in carbon partitioning and nitrogen metabolism, affecting starch biosynthesis, soluble sugars and free amino acid accumulation [32,33]. Additionally, altitude-related differences in soil properties and nutrient availability can modify mineral uptake and amino acid composition, contributing to variation in potato tuber nutritional profiles [26].

However, recent climate change is significantly impacting traditional practices [29,34,35].

In this context, the Matese plateau (province of Caserta, Campania region, Italy) is known for its characteristic potatoes, valued for their distinct flavour and texture [36]. Indeed, towns on the Matese plateau, including Alife, Castello del Matese, Gallo Matese, Prata Sannita and above all Letino, are renowned for their potatoes, highly appreciated by local communities as well as by Caserta and Naples citizens [37,38]. However, the cultivation of local potato landrace, known as ‘Matese black potato’ (in Italian ‘patata nera del Matese’), historically cultivated in the Matese area [37,39], has declined since local farmers replaced them with commercial varieties, such as Agria [40], Désirée [41] and Kennebec [42].

In this context, the present study was designed to investigate whether different commercial potato varieties (Agria, Désirée and Kennebec), when cultivated under the same pedoclimatic conditions of the Matese plateau, results in significant differences in their nutritional and biochemical composition. We hypothesised that variety-specific traits, interacting with the local environmental conditions, may influence proximate composition, free and total amino acid profiles, and antioxidant activity, thereby affecting the overall nutritional quality of the tubers.

## 2. Materials and Methods

### 2.1. Chemicals and Reagents

Folin–Ciocalteu reagent, *nor*-leucine(*nor*-Leu), gallic acid, ABTS [2,2′-azino-bis(3-ethylbenzothiazoline-6-sulfonic acid)], DPPH (2,2-diphenyl-1-picrylhydrazyl) and salts were obtained from Sigma-Aldrich Solutions (Merk Life Science, Milan, Italy). Chemicals and solvents for the Kjeldahl method were from Carlo Erba reagents (Milan, Italy), whereas those for automated amino acid analysis were provided from Biochrom (Cambridge, UK).

More details for specific reagents or enzymes are reported in the paragraphs below.

### 2.2. Potato Material and Growth Conditions

Five experimental sites for the cultivation of three different potato varieties (Agria, Désirée and Kennebec) were obtained from the Consorzio Agrario di Piedimonte Matese (Caserta, Italy) and cultivated across the Matese plateau. Table 1 reports the main characteristics of the tested varieties.

For each variety, tubers from the same certified lot were used and cultivated in five different experimental sites, located within the municipalities of Letino and Castello del Matese. Specifically, the Agria variety was grown at two sites (named Agria 1 and Agria 2) and the Désirée variety at two sites (named Désirée 1 and Désirée 2), while, due to seed availability and local agronomic practices, the Kennebec variety was cultivated at a single site. Each site represented distinct environmental and soil conditions. Meteorological conditions were considered homogeneous among all experimental sites, based on data recorded at the Letino weather station. The tubers were planted during the first ten days of May 2025. The soils were fertilised using organic nitrogen sources (farmyard manure). Agronomic practices included hoeing and hilling, without the use of herbicides. Finally, tubers were harvested in October 2025.

Fresh, pest-free, uniformly sized potatoes were selected for the trial, and all were stored at room temperature and protected from light. Information on sites where potatoes were collected are reported in Table 2. The potatoes without peels were cut into small portions (~5.0 g), freeze-dried using an Advantage LS 85 instrument (SP Scientific, Stone Ridge, NY, USA), ground into a fine powder and stored at −80 °C in plastic bags protected from light and humidity until further analyses.

### 2.3. Proximate Composition

The macronutrient content of potatoes was determined using standard AOAC protocols [43], specifically, the Kjeldahl method (AOAC 920.87; nitrogen factor used 6.25) for crude protein content and Soxhlet apparatus using CHCl_3_ as the extracting solvent (AOAC 948.22) for total lipid. The total carbohydrate content was obtained by subtracting the value of total ash, lipids and proteins from the total dry matter, following the FAO indications [44].

### 2.4. Ash Content and Moisture Content

Ash content and moisture levels of potato flour were determined according to the AOAC official method [43]. In particular, the AOAC methods for determining ash and moisture were AOAC 923.03 and AOAC 925.10, respectively.

### 2.5. Free and Total Amino Acid Composition

For the analysis of free amino acids, three aliquots of potato flour (~150 mg) were precipitated with 80% cold ethanol (1.0 mL) in the presence of *nor*-Leu (50 nmol) as an internal standard; homogenised with a Teflon pestle; and centrifuged at 14,000× *g*, 4 °C for 30 min. The supernatant was lyophilised, resuspended with 3.0% sulfosalicylic acid (500 mL) to precipitate any protein fraction still present, centrifuged again and analysed (see below).

For the analysis of total (free and protein) amino acids, aliquots of potato flour (~50 mg) were hydrolysed in glass tubes under vacuum with 0.5 mL of 6.0 M HCl containing 0.02% phenol and *nor*-Leu as an internal standard at 110 °C for 20 h. Following the hydrolysis, HCl was removed under vacuum, and samples were resuspended in 0.5 mL of 0.2 M lithium citrate buffer (pH 2.2).

Aliquots of hydrolysed and non-hydrolysed samples were directly analysed on a Biochrom30+ amino acid analyser (Biochrom, Cambridge, UK), equipped with a polyvinyl sulfonate cation exchange column for physiological fluids, a post-column ninhydrin derivatisation system and a two-channel detection system set at 570 and 440 nm, to allow proline and hydroxyproline detection [45]. Amino acid standard solutions were used to create a calibration curve within the following linearity range: 2.5; 5.0 and 10 nmol. The calibration curve was very good (R^2^ ≥ 0.996). The limits of detection (LOD) and quantification (LOQ) were 0.48 and 0.95, respectively.

### 2.6. Determination of Total Phenol Content and Antioxidant Activity

#### 2.6.1. Extraction of Total Phenols

Total phenols were extracted according to Landi et al. (2025) with some modifications [46]. Briefly, 1.0 g of potato flour was resuspended in 3.0 mL of methanol/water (80:20; v:v) and sonicated for 30 min at 25 °C using 70% amplitude. The extract obtained was centrifuged at 10,000× *g* for 30 min at 4 °C (Allegra V-15R (Beckman Coulter, Brea, CA, USA). The supernatant was collected and stored at −80 °C until further analysis.

#### 2.6.2. Determination of Total Phenol Content (TPC)

TPC of methanolic extract obtained as described in Section 2.6.1 was determined using the Folin–Ciocalteu method. TPC value was expressed as mg of gallic acid equivalents (GAE) per 100 g of fresh weight (FW) potatoes.

#### 2.6.3. Evaluation of Antioxidant Activities (AOX)

The ABTS^•+^ and DPPH^•^ radical cation scavenging capacity of methanolic extract obtained as described in Section 2.6.1 was estimated as previously reported [47]. The results were expressed as µmol Trolox^®^ Equivalents Antioxidant Capacity (TEAC) per 100 g of fresh weight (FW) potatoes.

### 2.7. Determination of Mineral Content

Aliquots of the powdered samples (~250 mg) were mineralised in an Ethos 900 microwave digestion system (Milestone™ Srl, Sorisole, Bergamo, Italy) endowed with temperature control, by a combination of hydrogen peroxide and nitric acid (H_2_O_2_ 50% *v*/*v*: HNO_3_ 65% *v*/*v* = 1:3). After digestion, the solutions were diluted by deionised water to a final volume of 50 mL. The nutrient (Ca, Fe, K, Mg, Mn, Na, P, Se and Zn) concentrations were quantified by atomic absorption spectrometry (SpectrAA 20 Varian, Mulgrave, Victoria, Australia) via flame furnace (FAAS) using standard solutions (STD Analyticals, Carlo Erba, Val de Reuil, France). Selenium (Se) was determined by graphite furnace atomic absorption spectrometry (GF-AAS) due to its trace-level concentration. Standard solutions were used to create calibration curves at five concentration levels within the following linearity ranges (mg/L): Ca (0.5–10), Fe (0.1–5), Mg (0.1–5), P (1–20), K (1–20), Na (0.2–10), Zn (0.05–2), Mn (0.02–2) and Se (0.01–0.5, GF-AAS). All the calibration curves were very good (R^2^ ≥ 0.997). Samples that were outside the calibration range were diluted appropriately before being analysed. The LOD and LOQ were calculated as 3σ and 10σ of the blank signal, respectively. This confirms the method’s high sensitivity, particularly for Se determination at µg/L levels. Accuracy was checked by analysis of standards (Resource Technology Corporation, Laramie, WY, USA), and the recovery was in a range of 90–110% for each element.

### 2.8. Statistical Analysis

For each variety and site, three independent biological samples were collected (n = 3 biological replicates). Each biological sample was analysed three times, and the means and standard deviations (SDs) of the experimental values are reported. Data analysis was carried out using Microsoft Excel 365 (Microsoft Corporation, Redmond, WA, USA). The results were analysed statistically using a two-way ANOVA test, followed by Tukey’s multiple comparison test by using the GraphPad Prism 8 software (GraphPad Software Inc., Boston, MA, USA). Significance was accepted at *p* < 0.05.

## 3. Results and Discussion

### 3.1. Proximate Composition Analysis

The nutritional composition of the different potatoes’ varieties (Agria, Desirée and Kennebec) grown on the Matese plateau (hereafter, Matese potatoes) is shown in Table 3. These data were compared with the nutritional values reported in the USDA database for “gold” potatoes, the latter used as a reference [48]. Significant differences were found in terms of moisture and total carbohydrate content, attributed to the different cultivation sites (Table 2) and the different varieties used. These findings may reflect not only the genotypic variability among potato varieties analysed but also the influence of the specific environmental conditions of the Matese plateau, including altitude, soil characteristics and temperature fluctuations, which are known to affect carbon allocation and water retention in tubers.

The nutritional analysis of Matese potatoes showed a protein content ranging from 1.98 to 3.07 g per 100 g of fresh weight (FW) potatoes. Among them, the Agria 1 and Désirée 1 varieties exhibited the highest total protein content (around 3.1 g per 100 g of FW potatoes, respectively). Considering this average value, the Kennebec variety showed a slightly lower but comparable protein content, while Agria 2 and Désirée 1 have protein contents 1.6- and 1.5-fold lower, respectively. A comparison with data reported in the USDA database for ‘gold’ potatoes (1.81 g of proteins per 100 g of FW potatoes) [48] highlighted that Agria 2 and Désirée 2 have a protein content comparable to the variety used as a reference, while the other samples analysed showed a protein content approximately 1.6-fold higher.

Furthermore, lipid content was less than 0.1 g per 100 g of FW potatoes in all analysed Matese potato varieties; this value was 2.6-fold lower than that of ‘gold’ potatoes (0.26 g per 100 g FW potatoes) used as a reference [48]. Carbohydrates were the most abundant macronutrient in all analysed Matese potato varieties, with values ranging from 12.05 to 15.78 g per 100 g of FW potatoes, consistent with the value reported for ‘gold’ potatoes (16 g per 100 g of FW), used as a reference [48]. Specifically, the Kennebec variety showed the highest total carbohydrate content (17.4%), followed by Agria 1 and-2 (~15.7%), as well as Désirée 1 and-2 (~12.6%). The Agria 1 and Kennebec varieties showed similar ash contents (~1.4 g per 100 g of FW), a value approximately 1.1-, 1.2- and 1.3-fold higher than those of Désirée 1, Agria 2 and Désirée 2, respectively. Moreover, the average ash content of all analysed Matese potato varieties was about 1.4-fold higher than that of ‘gold’ potatoes (0.89 g per 100 g of FW) [48].

The higher ash content observed in Matese potatoes may be linked to the slightly alkaline soil pH (7.2–7.9; Table 2), which can enhance the availability and uptake of minerals such as calcium and magnesium, thereby increasing the total mineral residue in tubers. In addition, the mineral-rich soils typical of mountainous areas may further contribute to this enrichment.

Finally, the total moisture content of all analysed Matese potato varieties ranged from 78.42 to 84.68%, matching the value reported for ‘gold’ potatoes (81%) [48]. In particular, the Kennebec variety had the lowest moisture content (~78%), followed by the two Agria samples (average value 80.43%) and two Désirée samples (average value 83.5%).

Overall, taking in consideration the average nutritional composition of all analysed varieties, the total protein, lipid and carbohydrate contents of the Matese potatoes were 2.6 g, 0.1 g and 14.8 g per 100 g of fresh weight (FW) potatoes, respectively. These data indicate that potatoes cultivated on the Matese plateau are low in calories, with an energy content of approximately 70.4 kcal per 100 g of FW potatoes.

### 3.2. Amino Acid Content and Protein Quality

Potato proteins are highly digestible and have a balanced amino acid profile, comparable to that of milk and egg proteins [49]. As reported in Table 4, the total amino acid composition of all analysed Matese potato varieties included all essential and non-essential amino acids, except tryptophan, which could not be determined under the acid hydrolysis conditions used, due to the methodological limitation. The obtained data revealed significant differences among the analysed Matese potato varieties, likely due to variations in cultivation sites. Data show that Glx (glutamic acid + glutamine; ranging from 179 to 221 mg per 100 g of FW) and Asx (aspartic acid + asparagine; ranging from 167 to 194 mg per 100 g of FW) were the most abundant amino acids in all analysed samples, accounting for about 17% and 15% of the total amino acid composition, respectively. These findings are consistent with those reported by Zhu et al. (2010), showing that Glx and Asx were the most abundant amino acids in the 16 potato varieties analysed [50]. In addition to these, other abundant amino acids include valine (ranging from 76.4 to 101.0 mg per 100 g of FW; ~8% of the total amino acid composition), arginine (ranging from 72.7 to 99.0 mg per 100 g of FW; ~7% of the total amino acid composition) and lysine (ranging from 66.1 to 97.0 mg per 100 g of FW; ~7% of the total amino acid composition), while the remaining amino acids ranged between 2 and 5% of the total. Furthermore, in absolute terms, considering the total amino acid content, the Agria 1 variety showed the highest value (1.4 g per 100 g of FW), followed by the Désirée 1 and Kennebec varieties (averagely 1.26 g per 100 g of FW), whereas the Agria 2 and Désirée 2 varieties exhibited lower values (averagely 1.07 g per 100 g of FW). Additionally, when only essential amino acids were considered, it was found that they represented an average of 38% of the total amino acid composition.

As shown in Table 5, the essential amino acid composition of Matese potato varieties was determined and expressed as mg per g of protein.

**Figure 1:**
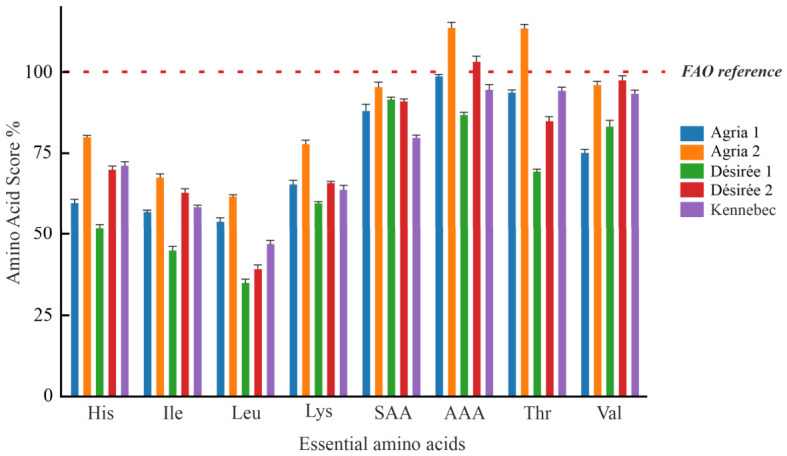
Chemical score of the essential amino acids obtained for different potato varieties grown on the Matese plateau.

Furthermore, the chemical score of all essential amino acids was calculated relative to the FAO reference protein [51] for each analysed sample, and the results are presented in Figure 1.

The data obtained show that Matese potato varieties have a high nutritional content of aromatic amino acids (AAA: phenylalanine + tyrosine) and threonine, with average chemical scores of ~99.8% and 91.6%, respectively. In particular, the Agria 2 variety achieved the highest chemical score for both AAA and threonine, reaching 114% in each case.

These findings are consistent with those reported by Galdòn et al. (2010), who found chemical scores for these amino acids up to 230% in different potato varieties grown in the Canary Islands [52]. Leucine was identified as the limiting amino acid in all analysed samples, with an average chemical score of ~48%. Specifically, the Désirée 1 and 2 varieties had the lowest leucine chemical scores (35% and 39%, respectively), the Agria 1 and Kennebec varieties had intermediate chemical scores (54% and 47%, respectively), while the Agria 2 variety had the highest score (60%). These results are in agreement with those reported by Bàrtova et al. (2015), who found that leucine is the limiting amino acid in potatoes cultivated in South America, with chemical scores ranging from 46 to 70% [53]. Finally, data on amino acid composition indicated that the Agria 2 and Agria 1 varieties had the highest protein quality scores (62 and 54%, respectively), while the Désirée 1, Désirée 2, and Kennebec varieties exhibited protein quality scores, respectively, 1.8, 1.6, and 1.3-fold lower than that of Agria 2.

### 3.3. Free Amino Acid Composition

Free amino acids are a key component of primary metabolism in potato tubers, serving as essential reservoirs and transporters of nitrogen. Some of these amino acids also act as precursors of flavour-related compounds formed during processing. In this context, the free amino acid profiles of all analysed Matese potato varieties were determined, and the resulting data are shown in Table 6.

In terms of total free amino acids, the Désirée 1 and Désirée 2 varieties had the highest contents (412 and 351 mg per 100 g of FW, respectively), followed by the Kennebec variety (271 mg per 100 g of FW), and the Agria 1 and Agria 2 varieties (219 and 202 mg per 100 g of FW, respectively). No statistically significant differences were observed among analysed samples, except for glutamine and asparagine.

**Figure 2:**
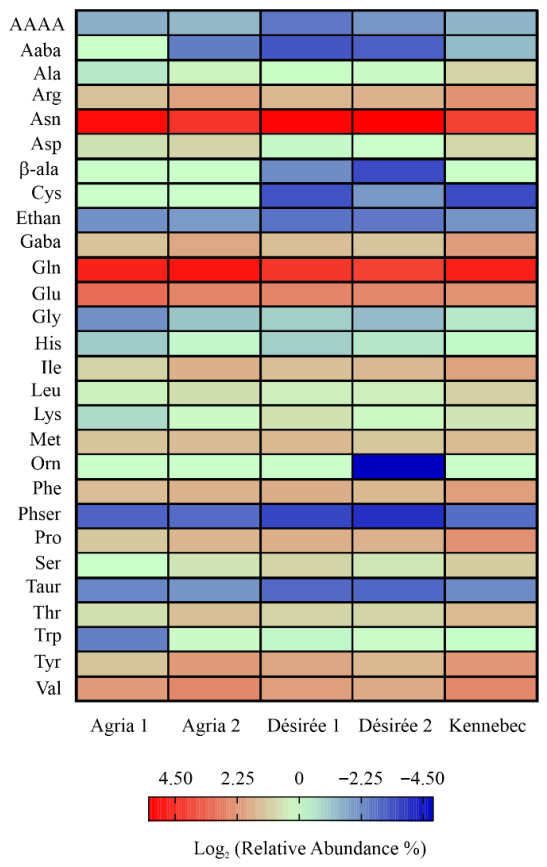
Heatmap showing the Log2 of the relative abundance percentage of free amino acids retrieved in the different potato varieties grown on the Matese plateau.

However, to emphasise the qualitative and quantitative differences among the analysed samples, a heat map was used to display the relative percentage abundance of the free amino acids (Figure 2). The graph shows that asparagine and glutamine were the most abundant free amino acids, which together account for ~50% of the total and are considered the predominant free amino acids in potatoes [54]. Furthermore, although these amino acids are important for plants as the main nitrogen transporters [55], high levels of asparagine in combination with reducing sugars can led to acrylamide formation during thermal processing of potatoes. Therefore, low levels of asparagine in potatoes may help reduce acrylamide production [56]. In this context, the asparagine levels of all analysed Matese potato varieties were compared with those of nine Italian potato varieties, which showed levels ranging from 15.46 to 458.46 mg per 100 g of FW [57], consistent with the levels observed in other potato samples. Notably, the Agria 1 and Agria 2 varieties contained ~1.8 and 3.7-fold less asparagine than the Agria Italian variety (146.65 mg per 100 g of FW) reported in the literature [57], while the Kennebec variety had an asparagine content similar to the Arinda Italian variety (50.34 mg per 100 g of FW) [57] reported in the literature. On the other hand, comparable asparagine levels were observed in the Désirée 1 and Désirée 2 varieties (averagely 156.10 mg per 100 g of FW), which were the highest among all analysed Matese potato varieties and comparable to the Merit Italian variety (146.65 mg per 100 g of FW) [57] reported in the literature. This observed variation in free asparagine is highly relevant from a food safety perspective, as this amino acid represents the main limiting precursor of acrylamide formation during high-temperature processing of potato products [58]. Therefore, differences in asparagine content directly affect acrylamide-forming potential. The mechanistic basis for this relationship lies in the central role of asparagine in nitrogen metabolism and its high reactivity in the Maillard reaction [59].

On the other hand, considering the remaining 50% of the total free amino acid composition (Figure 2), glutamic acid, valine and arginine were the most abundant amino acids after asparagine and glutamine. Glutamic acid ranged from 5 to 9% of the total free amino acids, valine from 4 to 6% and arginine from 2.5 to 5% of the total free amino acids across all analysed Matese potato varieties.

The remaining free amino acids were below 4%, except for proline (5.1% of the total free amino acids), which was ~1.8-fold higher in the Kennebec variety with respect to other analysed Matese potato varieties. Finally, seven non-protein amino acids were detected (Figure 2), with γ-aminobutyric acid (GABA) being the most abundant, ranging from 2.3 to 4.2% of total free amino acids, while the remaining free amino acids did not exceed 0.4% of the total. Notably, GABA is involved in numerous physiological processes in plants and is toxic to harmful insects, and it is typically found in high amounts in plant storage organs, such as potato tubers [60].

### 3.4. Total Phenolic Content (TPC) and Antioxidant Activity (AOX)

**Figure 3:**
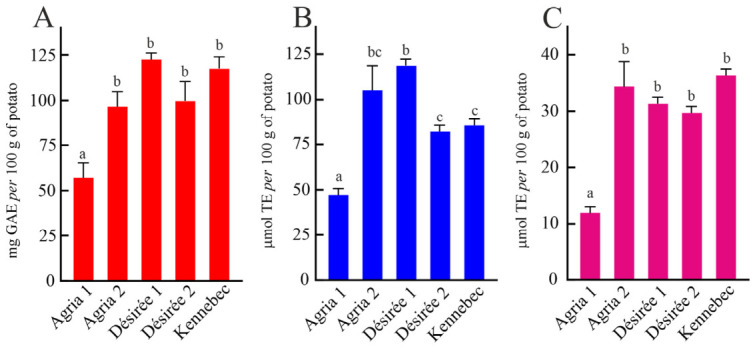
Total phenolic content (TPC) and antioxidant activity (AOX) of different potato varieties grown on the Matese plateau. (**A**) TPC was expressed as mg of gallic acid equivalents (GAE) per 100 g of fresh weight (FW) potatoes. (**B**) ABTS assay expressed as µmol of Trolox Equivalents (TE) per 100 g of FW potatoes. (**C**) DPPH assay expressed as µmol of TE per 100 g of FW potatoes. Different letters indicate statistically significant differences according to Tukey’s multiple comparison test (*p* < 0.05).

Potatoes represent an important dietary source of polyphenolic compounds exhibiting several bioactive properties, including antioxidant, anti-inflammatory and anti-tumour effects. Notably, these phytochemicals have been associated with lipid regulation, radioprotective effects and potential prevention of atherosclerosis [61]. In this context, to gain insight into their nutritional and functional properties, the total phenolic content (TPC) of all Matese potato varieties was analysed (Figure 3A).

TPC values ranged from 57.85 to 123.27 mg gallic acid equivalent (GAE) per 100 g of FW, corresponding to ~255.70–544.85 mg GAE per 100 g of dry weight (DW). The Désirée 1 variety showed the highest TPC (123.27 mg GAE per 100 g FW), which was ~2.1-fold higher than that of Agria 1 (the variety with the lowest TPC) and comparable to the other varieties. Furthermore, a comparison with literature data indicated that Matese potato varieties exhibit higher TPC than other reported varieties, whose TPC values range from 8.8 to 70.7 mg GAE per 100 g of DW [62]. These findings highlight that genuine phenolic enrichment occurs under Matese conditions. Furthermore, ABTS and DPPH assays were performed to evaluate the antioxidant activity (AOX) of the extracts from Matese potatoes (Figure 3B,C, respectively). A direct correlation between TPC and AOX was observed. In particular, the Agria 1 variety exhibited both the lowest TPC and the lowest AOX in both assays. Furthermore, Matese potato varieties exhibited ABTS radical scavenging activities ranging from 50 to 119 µmol of Trolox Equivalents (TE) per 100 g of FW, while DPPH radical scavenging activities ranged from 12 to 37 µmol TE per 100 g of FW. Notably, the Agria 2 and Désirée 1 varieties displayed more ABTS activity: ~2.3-fold higher than the Agria 1 variety and 1.3-fold higher than both the Désirée 1 and Kennebec varieties. These differences were not observed in DPPH radical scavenging activities, except for the Agria 1 variety, exhibiting values ~2.7-fold lower than those of other Matete potato varieties. Beyond the observed correlation between TPC and AOX, the divergent responses of ABTS and DPPH assays can be attributed to their distinct reaction mechanisms and differential sensitivity to specific phenolic classes. Indeed, ABTS assay reacts with a broad range of antioxidants through both electron-transfer and hydrogen-atom transfer mechanisms and is applicable in aqueous and organic media. Consequently, it efficiently detects hydrophilic and lipophilic phenolic compounds, including phenolic acids, flavonoid glycosides, anthocyanins and polymeric flavanols [63]. In contrast, the DPPH assay is more selective toward small, non-polar and sterically accessible antioxidants and often reacts slowly or incompletely with high-molecular-weight or highly substituted phenolic compounds. As a result, DPPH may underestimate the AOX in complex plant matrices characterised by diverse and polymeric phenolic profiles [64]. Therefore, variety-dependent qualitative differences in phenolic composition are more effectively resolved by ABTS assay compared with DPPH. These findings highlight the importance of using complementary antioxidant assays to achieve a more comprehensive evaluation of the antioxidant potential of potato varieties exhibiting distinct phenolic fingerprints.

### 3.5. Mineral Content

To further characterise the nutritional profile of Matese potato varieties, the mineral content was analysed (Table 7). Significant differences were observed among the five Matese potato varieties for most minerals, except for iron, sodium, zinc, selenium and manganese.

These results suggest that mineral accumulation in the analysed tubers is influenced by both genetic factors (varietal differences) and environmental conditions, particularly soil mineral availability and pedoclimatic characteristics of the Matese plateau.

Potato tubers are among the few vegetables rich in essential alkaline minerals such as potassium, magnesium and calcium. These minerals are crucial for maintaining acid–base balance, supporting energy metabolism and ensuring normal neuromuscular function [65]. These analysed potatoes contained high levels of potassium, ranging from 491 to 559 mg per 100 g of FW, consistent with previously reported data for other potato varieties, which exhibit potassium contents ranging from 380 to 545 mg per 100 g [66]. From a nutritional perspective, the high potassium content is particularly relevant, as increased dietary potassium intake has been associated with improved cardiovascular health and reduced risk of hypertension. Moreover, the variability observed among varieties may reflect differences in potassium uptake efficiency and storage capacity at the tuber level [67]. Furthermore, Matese potato varieties contained phosphorus, calcium and magnesium at concentrations ranging from 46.0 to 54.0 mg, 28.2 to 30.4 mg and 22.1 to 27.0 mg per 100 g of FW, respectively. In addition, these elements were present at higher concentrations in Matese potato varieties compared to other varieties, which exhibited maximum levels of 53.9 mg phosphorus, 16.5 mg calcium and 13.1 mg magnesium per 100 g [66]. On the other hand, the remaining minerals, such as sodium (averagely 5.9 mg per 100 g of FW), iron (averagely 3.7 mg per 100 g of FW), zinc (averagely 0.37 mg per 100 g of FW) and manganese (averagely 0.15 mg per 100 g of FW), accounted for only 1.6% of the total mineral content. Finally, the selenium content of Matese potato varieties ranged from 0.45 to 0.70 µg per 100 g of FW, consistent with values reported in the USDA database for ‘gold’ potatoes, which contain less than 2.5 µg per 100 g of FW [48]. This relatively low selenium concentration is consistent with the generally low availability of this element in most agricultural soils and confirms that potatoes are not a major dietary source of selenium. Overall, the results highlight that Matese potato varieties represent a valuable source of essential microminerals, particularly potassium, magnesium, calcium and phosphorus. The observed differences among varieties, combined with their favourable mineral profile, suggest a potential for their valorisation in terms of both nutritional quality and market differentiation.

## 4. Conclusions

This study provides a comparative analysis of the Agria, Désirée and Kennebec potato varieties grown on the Matese plateau, focusing on their nutritional composition, amino acid profiles and antioxidant activity. Despite their widespread cultivation in this area, limited information was previously available on their nutritional and biochemical characteristics under Matese plateau conditions.

Significant differences were observed among varieties in protein, carbohydrate and moisture content, while lipids and ashes levels were comparable among the varieties.

The amino acid profile was generally balanced, with all essential amino acids present; Glx and Asx were predominant, followed by valine, arginine and lysine. Essential amino acids accounted for approximately 38% of the total amino acids, while leucin was identified as the limiting amino acid. Asparagine and glutamine accounted for ~50% of total free amino acids, with Agria varieties showing lower asparagine levels, beneficial for reducing acrylamide formation.

Matese potato varieties showed significant antioxidant activity, reflecting their high polyphenol content, while ABTS and DPPH assays correlated with TPC confirm that potatoes cultivated on Matese plateau are a rich dietary source of bioactive compounds with antioxidant potential. In addition, they contained relevant levels of essential minerals like potassium, phosphorus, calcium and magnesium, confirming their nutritional value. Overall, potatoes cultivated on the Matese plateau represent a nutritionally valuable food, characterised by high-quality proteins, bioactive compounds and favourable mineral compositions. These findings support their potential role in promoting local consumption, enhancing cultivation and contributing to the valorisation of regional food systems and agri-food tourism.

## Figures and Tables

**Table 1:** Main characteristics of potato varieties cultivated across the Matese Plateau.

	*Potato Varieties*		
	*Agria*	*Désirée*	*Kennebec*
*Utility type*	Processing/Fresh market	Fresh market	Fresh market/Multipurpose
*Earliness*	Medium-late	Medium-early	Medium-late
*Plant vigour*	High	Medium to high	High
*Tuber characteristics*	Oval/long-oval, yellow skin	Oval, red skin	Oval to irregular, light skin
*Flesh colour*	Deep yellow	Light yellow	White
*Main quality traits*	High dry matter content; excellent frying quality	Good cooking quality; versatile culinary use	Mealy texture; good frying and boiling quality

**Table 2:** Geographic location, altitude and soil characteristics of the sites where different potato varieties were grown and harvested.

Sample	Longitude and Latitude	m. a. s. l. *	Soil Texture	Soil pH
Agria 1	14°18′42.8″ E 41°26′44.7″ N	1190	sandy clay	7.4
Agria 2	14°22′21.1″ E 41°25′30.1″ N	1095	sandy	7.2
Désirée 1	14°15′20.0″ E 41°27′05.3″ N	932	clayey, sandy, silty	7.3
Désirée 2	14°25′34.5″ E 41°22′25.7″ N	887	sandy clay	7.9
Kennebec	14°14′38.1″ E 41°26′59.3″ N	957	sandy clay	7.5

* m. a. s. l., metres above sea levels.

**Table 3:** Nutritional values of different potato varieties grown on the Matese plateau. Values are means (±SD) of triplicate analyses (*n* = 3) and are expressed as g per 100 g on a fresh weight basis (FW).

	Agria 1	Agria 2	Désirée 1	Désirée 2	Kennebec
Proteins	3.07 ± 0.42 a	1.98 ± 0.03 b	3.02 ± 0.15 a	2.08 ± 0.03 b	2.68 ± 0.13 a
Lipids	<0.1 a	<0.1 a	<0.1 a	<0.1 a	<0.1 a
Ash	1.39 ± 0.15 a	1.19 ± 0.04 a	1.28 ± 0.04 a	1.10 ± 0.06 a	1.38 ± 0.04 a
Moisture	79.67 ± 0.82 a	81.20 ± 1.11 b	82.35 ± 1.24 b	84.68 ± 0.07 c	78.42 ± 1.18 a
Carbohydrates	15.78 ± 0.55 a	15.54 ± 1.17 a	13.25 ± 0.20 b	12.05 ± 0.04 b	17.42 ± 0.26 c

In each row, different letters indicate statistically significant differences according to Tukey’s multiple comparison test (*p* < 0.05).

**Table 4:** Total amino acid compositions of different potato varieties grown on the Matese plateau. Values are means (±SD) of triplicate analyses (*n* = 3) and are expressed on a fresh weight basis (mg/100 g).

Amino Acid	Agria 1	Agria 2	Désirée 1	Désirée 2	Kennebec
*essential amino acids*					
His	29.51 ± 0.11 a	25.44 ± 0.24 a	25.19 ± 0.80 a	23.42 ± 0.21 a	30.73 ± 0.52 a
Ile	52.82 ± 1.59 a	40.36 ± 0.46 b	41.06 ± 1.04 b	39.48 ± 1.04 b	47.26 ± 0.19 ab
Leu	101.73 ± 1.10 a	74.99 ± 1.79 b	65.21 ± 2.54 c	50.23 ± 2.87 d	77.24 ± 3.76 b
Lys	97.05 ± 2.24 a	74.39 ± 0.72 bd	86.93 ± 4.98 c	66.15 ± 0.85 b	82.58 ± 3.29 dc
Met	32.02 ± 2.22 a	23.44 ± 0.04 b	34.85 ± 1.87 a	24.41 ± 0.00 b	27.68 ± 1.61 ab
Phe	79.71 ± 0.77 a	56.78 ± 0.28 b	61.13 ± 1.68 b	47.00 ± 0.59 c	65.20 ± 0.50 b
Thr	72.26 ± 0.57 a	56.41 ± 0.68 bd	52.72 ± 1.66 bc	44.37 ± 0.93 c	63.42 ± 1.38 d
Trp	n.d.	n.d.	n.d.	n.d.	n.d.
Val	92.74 ± 0.94 a	76.43 ± 0.68 b	101.05 ± 4.33 a	81.47 ± 1.85 b	100.54 ± 0.11 a
*non-essential amino acids*					
Ala	62.34 ± 1.02 a	46.66 ± 0.27 bc	43.09 ± 1.79 b	34.13 ± 0.20 d	53.23 ± 1.85 c
Arg	86.91 ± 3.58 a	72.66 ± 1.82 bc	77.72 ± 3.01 be	80.03 ± 1.40 ace	99.00 ± 3.16 d
Asx	188.39 ± 5.58 a	166.50 ± 4.39 b	194.23 ± 13.77 a	170.48 ± 6.75 bc	178.56 ± 8.84 c
Cys ^§^	30.46 ± 0.91 a	20.20 ± 2.48 bc	19.35 ± 1.45 ae	21.71 ± 2.12 c	29.07 ± 0.09 bce
Glx	220.91 ± 7.25 ac	182.86 ± 3.73 b	223.09 ± 14.52 a	179.66 ± 5.11 b	213.53 ± 10.27 c
Gly	59.75 ± 1.22 a	44.31 ± 0.72 b	39.30 ± 1.84 b	30.08 ± 0.52 c	46.20 ± 1.69 b
Pro	73.76 ± 0.60 a	51.97 ± 0.58 b	51.34 ± 2.85 c	39.43 ± 0.03 b	62.49 ± 0.52 d
Ser	79.05 ± 1.36 a	56.58 ± 0.25 b	65.07 ± 2.90 bd	46.53 ± 0.69 c	66.32 ± 3.53 d
Tyr	45.05 ± 10.90 a	35.82 ± 2.62 bc	46.92 ± 3.32 a	41.36 ± 1.22 ac	39.19 ± 6.64 ac
**Total (g/100 g)**	**1.40**	**1.11**	**1.24**	**1.02**	**1.27**

In each row, different letters indicate statistically significant differences according to Tukey’s multiple comparisons test (*p* < 0.05). n.d., not determined. ^§^ Cys amount was evaluated after performic acid oxidation. Asx and Glx represent the sum of aspartic acid + asparagine and glutamic acid + glutamine, respectively, due to the acid hydrolysis process, which converts amide forms into their corresponding acids.

**Table 5:** Amino acid composition (mg/g protein) of different potato varieties grown on the Matese plateau and comparison with FAO/WHO reference patterns for adults.

	Agria 1	Agria 2	Désirée 1	Désirée 2	Kennebec	*FAO Reference ^#^*
His	9.61	12.85	8.34	11.26	11.47	16.00
Ile	17.20	20.38	13.60	18.98	17.63	30.00
Leu	33.14	37.87	21.59	24.15	28.82	61.00
Lys	31.61	37.57	28.79	31.80	30.82	48.00
SAA *	20.35	22.04	21.17	21.04	18.43	23.00
AAA ^$^	40.64	46.77	35.78	42.48	38.95	41.00
Thr	23.54	28.49	17.46	21.33	23.67	25.00
Val	30.21	38.60	33.46	39.17	37.51	40.00

^#^ Amino acid scoring pattern for adults (mg/g protein) recommended by the FAO/WHO [51]; * SAA = (Met + Cys); ^$^ AAA = (Phe + Tyr).

**Table 6:** Free amino acid compositions of different potato varieties grown on the Matese plateau. Values are means (±SD) of triplicate analyses (*n* = 3) and are expressed on a fresh weight basis (mg/100 g).

Free Amino Acid	Agria 1	Agria 2	Désirée 1	Désirée 2	Kennebec
***protein amino acids***					
Ala	1.62 ± 0.05 a	2.42 ± 1.03 a	4.30 ± 0.38 a	3.75 ± 0.94 a	5.14 ± 0.02 a
Arg	5.42 ± 0.01 a	7.98 ± 1.36 a	11.79 ± 0.86 a	10.89 ± 0.34 a	13.56 ± 0.04 a
Asn	79.25 ± 0.01 a	39.63 ± 0.64 b	157.99 ± 13.63 c	154.21 ± 81.15 c	44.80 ± 0.49 b
Asp	3.31 ± 0.02 a	3.76 ± 0.21 a	3.74 ± 0.44 a	3.53 ± 0.45 a	4.89 ± 0.07 a
Cys	n.d.	n.d.	0.41 ± 0.00 a	0.90 ± 0.41 a	0.25 ± 0.00 a
Gln	57.09 ± 1.36 a	62.71 ± 7.08 ac	78.49 ± 9.00 bc	58.06 ± 2.53 ad	73.28 ± 9.09 bcd
Glu	19.12 ± 0.00 a	12.26 ± 0.18 a	25.28 ± 2.18 a	20.27 ± 4.82 a	13.46 ± 0.52 a
Gly	0.50 ± 0.01 a	0.93 ± 0.37 a	2.18 ± 0.22 a	1.43 ± 0.52 a	2.00 ± 0.00 a
His	1.09 ± 0.01 a	1.78 ± 0.10 a	2.16 ± 0.25 a	2.56 ± 0.29 a	2.45 ± 0.15 a
Ile	4.12 ± 0.00 a	6.47 ± 1.18 a	10.36 ± 0.75 a	9.79 ± 1.52 a	10.47 ± 0.05 a
Leu	2.69 ± 0.00 a	3.23 ± 0.65 a	5.19 ± 0.36 a	4.44 ± 0.47 a	5.43 ± 0.03 a
Lys	1.33 ± 0.00 a	2.18 ± 0.40 a	6.38 ± 0.56 a	3.89 ± 1.29 a	4.00 ± 0.00 a
Met	5.17 ± 0.02 a	5.40 ± 0.58 a	11.59 ± 0.96 a	7.80 ± 1.54 a	7.43 ± 0.02 a
Phe	5.77 ± 0.02 a	6.24 ± 1.39 a	13.58 ± 1.04 a	9.90 ± 2.84 a	11.29 ± 0.06 a
Pro	4.83 ± 0.03 a	5.94 ± 2.32 a	13.29 ± 1.08 a	10.82 ± 2.64 a	13.85 ± 0.01 a
Ser	2.22 ± 0.03 a	2.97 ± 1.01 a	7.66 ± 0.87 a	5.06 ± 1.81 a	5.72 ± 0.09 a
Thr	3.42 ± 0.01 a	5.14 ± 0.79 a	7.76 ± 0.82 a	6.63 ± 0.90 a	7.49 ± 0.05 a
Trp	0.40 ± 0.13 a	2.15 ± 0.21 a	3.54 ± 0.13 a	3.68 ± 1.42 a	2.62 ± 0.08 a
Tyr	5.08 ± 0.03 a	8.90 ± 1.05 a	15.25 ± 1.32 a	10.17 ± 1.55 a	12.42 ± 0.01 a
Val	9.52 ± 0.06°	11.72 ± 1.61 a	17.28 ± 1.51 a	12.42 ± 0.11 a	15.93 ± 0.05 a
*non-protein amino acids* *					
AAAA	0.77 ± 0.04 a	0.78 ± 0.04 a	0.68 ± 0.17 a	0.87 ± 0.12 a	1.02 ± 0.13 a
Aaba	n.d.	0.36 ± 0.01 a	0.44 ± 0.01 a	0.42 ± 0.17 a	1.09 ± 0.00 a
β-ala	n.d.	n.d.	0.90 ± 0.10 a	0.33 ± 0.18 a	n.d.
Ethan	0.51 ± 0.00 a	0.53 ± 0.07 a	0.64 ± 0.05 a	0.58 ± 0.07 a	0.66 ± 0.01 a
GABA	5.20 ± 0.01 a	7.31 ± 1.67 a	10.49 ± 0.93 a	8.15 ± 1.28 a	11.46 ± 0.07 a
Orn	n.d.	n.d.	n.d.	0.12 ± 0.01	n.d.
Phser	0.28 ± 0.00 a	0.28 ± 0.03 a	0.36 ± 0.03 a	0.23 ± 0.03 a	0.39 ± 0.00 a
Taur	0.44 ± 0.04 a	0.49 ± 0.03 a	0.57 ± 0.07 a	0.46 ± 0.10 a	0.58 ± 0.01 a
**Total**	**219.13**	**201.57**	**412.31**	**351.33**	**271.70**

Three-letter codes have been used; n.d., not detected. In each row, different letters indicate statistically significant differences according to Tukey’s multiple comparisons test (*p* < 0.05). n.d., not detected. * Abbreviation: **AAAA**, L-α-aminoadipic acid; **Aaba**, α-aminobutyric acid; **β-ala**, β-alanine; **Ethan**, ethanolamine; **GABA**, γ-aminobutyric acid; **Orn**, ornitine; **Phser**, phosphoserine; **Taur**, taurine.

**Table 7:** Mineral content of different potato varieties grown on the Matese plateau. Values are the means (±SD) of triplicate analyses (*n* = 3) expressed on a fresh weight basis (mg/100 g), except Se, which is expressed on a µg/100 g of FW basis.

Element	Agria 1	Agria 2	Désirée 1	Désirée 2	Kennebec
**Ca**	30.40 ± 1.01 a	30.18 ± 0.30 a	28.25 ± 1.09 a	28.43 ± 0.09 a	29.77 ± 0.25 a
**Fe**	3.93 ± 0.28 a	3.75 ± 0.11 a	3.69 ± 0.04 a	3.61 ± 0.07 a	3.65 ± 0.09 a
**Mg**	27.00 ± 1.80 a	25.83 ± 0.42 ac	23.14 ± 0.16 b	22.08 ± 0.11 b	24.11 ± 0.17 bc
**P**	53.67 ± 1.53 a	52.33 ± 1.16 ac	48.60 ± 0.53 b	45.97 ± 0.09 d	50.33 ± 0.58 bc
**K**	559.30 ± 4.04 a	542.30 ± 2.52 b	499.67 ± 1.53 c	491.00 ± 2.65 d	520.50 ± 0.50 e
**Na**	6.07 ± 0.21 a	6.43 ± 0.12 a	5.52 ± 0.02 a	6.01 ± 0.03 a	5.00 ± 0.03 a
**Zn**	0.42 ± 0.01 a	0.42 ± 0.03 a	0.34 ± 0.01 a	0.32 ± 0.01 a	0.35 ± 0.01 a
**Se**	0.70 ± 0.01 a	0.61 ± 0.02 a	0.45 ± 0.01 a	0.40 ± 0.01 a	0.51 ± 0.01 a
**Mn**	0.20 ± 0.01 a	0.18 ± 0.01 a	0.15 ± 0.02 a	0.13 ± 0.01 a	0.15 ± 0.01 a

In each row, different letters indicate statistically significant differences according to Tukey’s multiple comparisons test (*p* < 0.05).

## Data Availability

The original contributions presented in the study are included in the article, further inquiries can be directed to the corresponding authors.
